# Import of global high-risk clones is the primary driver of carbapenemase-producing Pseudomonas aeruginosa in Norway

**DOI:** 10.1099/jmm.0.001944

**Published:** 2025-01-06

**Authors:** Bjørg Christina Haldorsen, Ørjan Samuelsen, Jessin Janice, Miriam Sare, Mari Molvik, Arnfinn Sundsfjord, The Norwegian Study Group on CP-Pa, Torunn Pedersen

**Affiliations:** 1 Norwegian Centre for Detection of Antimicrobial Resistance, Department of Microbiology and Infection Control, University Hospital of North Norway, Tromsø, Norway; 2 Department for Infection Control and Preparedness, Norwegian Institute of Public Health, Oslo, Norway; 3 Department of Medical Biology, Faculty of Health Sciences, UiT – The Arctic University of Norway, Tromsø, Norway

**Keywords:** carbapenemase-producing, global high-risk clones, molecular epidemiology, *Pseudomonas aeruginosa*

## Abstract

**Introduction.** Infections by carbapenemase-producing *Pseudomonas aeruginosa* (CP-Pa) are concerning due to limited treatment options. The emergence of multidrug-resistant (MDR) high-risk clones is an essential driver in the global rise of CP-Pa.

**Hypothesis/Gap Statement.** Insights into the molecular epidemiology of CP-Pa are crucial to understanding its clinical and public health impact. Despite the low incidence of infections in Norway, global spread requires an understanding of regional dissemination patterns.

**Aim.** This study aimed to investigate the phenotypic and genotypic characteristics of CP-Pa isolates in Norway and molecular epidemiology by utilizing available metadata.

**Methodology.** The study collection comprised all verified CP-Pa isolated in Norway from 2006 to 2022 (*n*=67) obtained from clinical (75%; *n*=50) or screening samples (22%; *n*=15) or had no available information (3%; *n*=2). Phenotypic analyses included antimicrobial susceptibility testing against clinically relevant antipseudomonal antibiotics and comparative testing for carbapenemase production using three different methods (*β*-CARBA, IMI/IMD gradient test and Coris O.K.N.V.I RESIST-5). Whole-genome sequencing was performed to identify virulence factors, resistance determinants and genomic relatedness.

**Results.** The isolates were categorized as MDR (*n*=39) encoding Verona integron-encoded metallo-*β*-lactamase (VIM) (*n*=28), New Delhi metallo-*β*-lactamase (NDM) (*n*=6), imipenemase metallo-*β*-lactamase (IMP) (*n*=4) or Guiana extended spectrum metallo-*β*-lactamase (*n*=1) carbapenemases or extensively drug-resistant (XDR; *n=*28) encoding VIM (*n*=11), NDM (*n*=9) or IMP (*n*=8) carbapenemases. CP-Pa numbers ranged from 1 to 7 annually, peaking at 17 in 2022. Most isolates (*n*=64) were associated with international travel or hospitalization abroad. Phylogenetic analyses identified nine clusters of closely related genomes, with one suspected case of domestic patient-to-patient transmission. Among 21 detected sequence types, several were global high-risk clones, including ST235 (*n*=12), ST111 (*n*=9), ST773 (*n*=9), ST253 (*n*=3), ST357 (*n*=3), ST395 (*n*=3), ST823 (*n*=3), ST233 (*n*=2), ST654 (*n*=2), ST260 (*n*=1) and ST308 (*n*=1), covering 72% of the Norwegian isolates. ST1047 (IMP-1) and ST773 (NDM-1) were associated with Ukrainian war victims. Carbapenemase detection rates for phenotypic tests were 88% (*β*-CARBA), 91% (IMI/IMD) and 94% (Coris) in our collection.

**Conclusion.** The study highlights the low incidence yet high genomic diversity of CP-Pa in Norway and the dominance of high-risk clones linked to imports, contributing to the high proportion of XDR.

## Introduction

The emergence of multidrug-resistant (MDR) pathogens in healthcare facilities worldwide poses a significant global threat to modern medical practices. *Pseudomonas aeruginosa* mainly colonizes vulnerable patients, leading to respiratory tract, bloodstream, wounds and complicated urinary tract infections [1,3]. *P. aeruginosa* is intrinsically resistant to several commonly used antibiotics [4,5] and has developed adaptive resistance strategies, such as chromosomal mutations mediating the downregulation of outer membrane proteins, upregulation of efflux pumps and increased AmpC expression [6]. Additionally, it can acquire resistance genes horizontally, including those encoding carbapenemases, 16S rRNA methylases and aminoglycoside-modifying enzymes (AMEs) [7,8].

Carbapenems are commonly used first-line antibiotics for severe infections, but their success is being compromised by the global rise of carbapenemase-producing *P. aeruginosa* (CP-Pa). The most prevalent carbapenemases in *P. aeruginosa* belong to the metallo-*β*-lactamases (Ambler class B) such as imipenemase metallo-*β*-lactamase (IMP), Verona integron-encoded metallo-*β*-lactamase (VIM) and New Delhi metallo-*β*-lactamase (NDM), as well as carbapenemase variants of Guiana extended spectrum metallo-*β*-lactamase (GES) (e.g. GES-5) and Klebsiella pneumoniae carbapenemase (Ambler class A) [8,11]. These enzymes are often linked to global high-risk clones [12,13]. Other carbapenemases, like Florence imipenemase, German imipenemase and Sao Paulo metallo-*β*-lactamase, are rare and typically related to specific genetic lineages with limited regional spread [12,14].

Global high-risk clones of *P. aeruginosa* consist of several successful genetic lineages, with ST235 and ST111 being the most widespread [13]. Despite being phylogenetically diverse, these high-risk clones share common characteristics, such as acquired *β*-lactamases, MDR/extensively drug-resistant (XDR) phenotypes and increased virulence [12,15, 16]. When resistance extends to all high-efficacy and low-toxicity agents, including carbapenems and fluoroquinolones, they are classified as difficult-to-treat resistant clones [17]. The widespread transmission and lack of effective therapies pose significant public health concerns [18]. Understanding the factors driving the persistence and dissemination of these clones is crucial for developing effective preventive measures.

In the Nordic countries, the prevalence of carbapenemase-producing Gram-negative bacteria is low compared to other regions and is often linked to imports [19,23]. Data from the European Antimicrobial Resistance Surveillance Network indicates that the percentage of carbapenem-resistant *P. aeruginosa* infections in Norway has varied from 1 to 6.8 since 2005 (https://atlas.ecdc.europa.eu/public/index.aspx), which is similar to other Northern European countries [24]. In Norway, these figures mainly reflect resistance due to adaptive chromosomal mutations, with the first CP-Pa isolate identified in 2006 [25]. The purpose of the current study was to analyse the molecular epidemiology associated with the emergence of CP-Pa in Norway from 2006 to 2022.

## Methods

### Strain collection

Our study collection consists of all confirmed CP-Pa isolates identified in Norway from 2006 to 2022 by the National Reference Laboratory (Table S1, available in the online version of this article). This collection includes 67 non-duplicate isolates submitted from 17/21 clinical microbiology laboratories across all 4 health regions. Isolates were obtained from various clinical specimens and screening samples processed by hospital laboratories for routine diagnostics. These were submitted for carbapenemase verification according to the Nordic Committee on Antimicrobial Susceptibility Testing guidelines (www.nordicast.org). We included one isolate per patient per year, selecting clinical isolates in cases of duplicates [identical sequence type (ST) and carbapenemase]. Reporting CP-Pa to the Norwegian Surveillance System for Communicable Diseases (MSIS) became mandatory in 2012. A case was defined as a patient positive for one or more CP-Pa within 1 year (https://www.fhi.no/publ/informasjonsark/meldingskriterier-for-sykdommer-i-msis/). Upon verification as CP-Pa, the responsible physician has to submit relevant patient data to MSIS through a specific questionnaire (https://www.fhi.no/publ/skjema/msis-meldingsskjema-nominativ-melding/), which includes indications for the laboratory examination (including contact with healthcare abroad), presumed site of infection [Norway (region), abroad (country) or unknown] and the likely site of exposure (including healthcare institutions). For each isolate included in the study collection, metadata regarding clinical infection, screening, foreign travel (import), age and sex (female, male or unknown) were retrieved from laboratory requisition forms and MSIS from 2012 onwards (https://www.fhi.no/en/ou/msis/).

### Phenotypic and genotypic analyses

Species identification was performed using matrix-assisted laser desorption/ionisation time-of-flight mass spectrometry (Bruker Daltonic Gmbh) and whole-genome sequencing (WGS). Antimicrobial susceptibility testing was conducted by broth microdilution using custom-made microtitre plates (TREK Diagnostic Systems/Thermo Fisher Scientific) and by disc diffusion for cefiderocol (Liofilchem). Results were interpreted according to EUCAST guidelines v.14.0 [26]. Initial PCR testing identified *bla*_IMP_, *bla*_VIM_, *bla*_NDM_ and *bla*_GES_ carbapenemase-encoding genes [27,28]. Carbapenemase activity/presence was further investigated phenotypically using the *β*-CARBA test (Bio-Rad), imipenem/EDTA combination strip (IMI/IMD, Liofilchem) and O.K.N.V.I RESIST-5 immunochromatography (Coris BioConcept), following the manufacturer’s instructions.

### WGS and analyses

Total DNA was extracted using the automated EZ1 platform (Qiagen). Paired-end libraries were generated using Nextera®/Illumina DNA Prep kits and sequenced on the NextSeq 550 or MiSeq platforms (Illumina). Quality control of sequences and assemblies was conducted using FastQC v.0.11.9 and Quast v.5.2.0. Reads were quality trimmed, and adapters were removed using Trimmomatic-0.35) and assembled with SPAdes v.3.12.0 [29]. Assemblies were annotated using Prokka v.1.14.0. Taxonomic classification was performed on reads and contigs using Kraken v.1.1, and multilocus sequence types (MLSTs) were identified using mlst v.2.23.0 (https://github.com/tseemann/mlst) by comparison to the PubMLST database (https://pubmlst.org/) [30]. Resistance genes and mutations were identified using AMRFinderPlus v.3.11.2 with database v.2023-08-08.2, applying a minimum identity and coverage of 90%. O-antigen serotypes were determined *in silico* by the *Pseudomonas aeruginosa* serotyper (PAst) program [31] and the presence of *exoU*/*exoS* by blast search. For core-genome (cg) MLST, the public scheme (https://www.cgmlst.org/ncs/schema/Paeruginosa3715/) with default minimum spanning tree (MST) cluster distance threshold (12 alleles) was used. Phylogenies were constructed using Ridom SeqSphere+ (https://www.ridom.de/seqsphere/) or Parsnp v.1.2 with the ‘–c’-flag and visualized with FigTree (http://tree.bio.ed.ac.uk/software/figtree/). Metadata coupling was facilitated using Phandango [32].

## Results

### Characteristics of the CP-Pa study collection

**Fig. 1.**
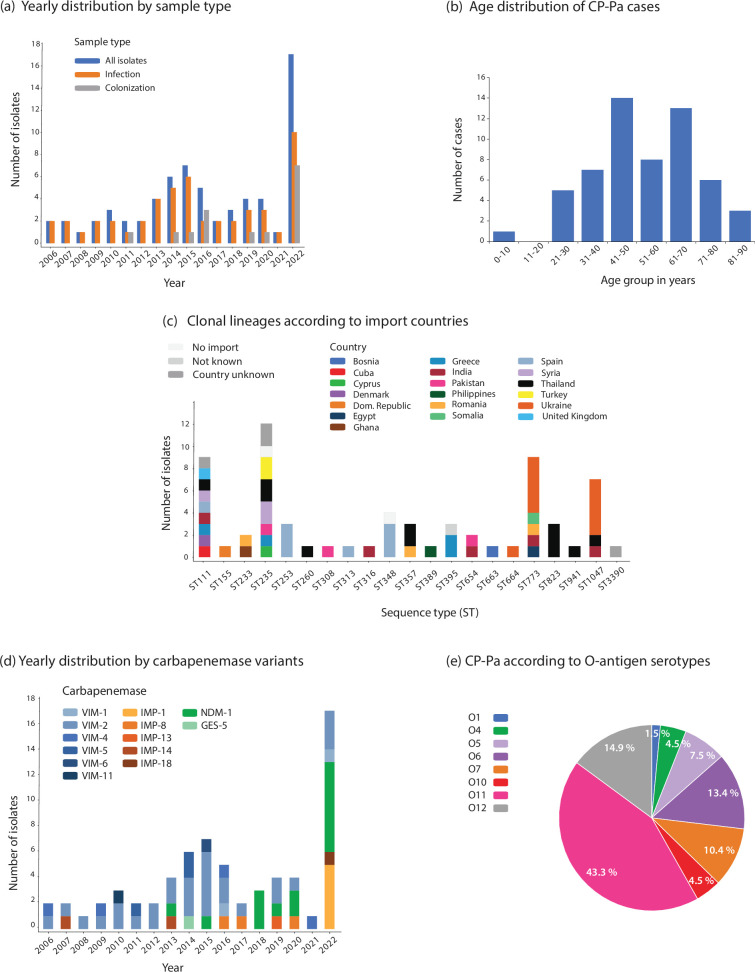
Distribution of CP-Pa isolates or cases in Norway from 2006 to 2022 according to (a) sample type by year of isolation, (**b**) patients’ age categories, (**c**) clonal lineages (ST) by import countries, (**d**) carbapenemase variants by year of isolation and (e) O-antigen serotypes.

A total of 67 CP-Pa isolated from 67 patients were included (Table S1), with annual numbers ranging from 1 to 17 (Fig. 1a). Of these, 75% (*n*=50) were clinical isolates obtained from blood (*n*=2), respiratory tract (*n*=7), urine (*n*=12), wound (*n*=13), other (*n*=15) or unknown site (*n*=1). For two of the isolates (3%), it was unknown whether they were from clinical or screening samples. The remaining isolates (*n*=15; 22%) were screening samples primarily obtained from rectal swabs (*n*=11), with a noticeable increase in 2022 (*n*=7). The median age of the patients was 52 years (range: 4–86 years). Among cases with a known gender (data missing for collection dates before 2011), 81% (46/57) were men. When categorized into age groups (Fig. 1b), most cases were found among patients aged 41–50 (*n*=14; 25%) and 61–70 (*n*=13; 23%). However, the small sample size undermines the statistical significance of these results, particularly for subgroups.

### Global high-risk clones associated with travel or hospitalization abroad

One of the primary questions in our study was the likely geographical origin of the CP-Pa isolates. The patient data retrieved from MSIS and laboratory requisition forms included information regarding the presumed origin, Norway, abroad (name of country/unknown country) or unknown, and the likely site of exposure (including healthcare institutions), revealing that most isolates (*n*=64; 96%) were linked to international travel or hospitalization abroad (Table S1). For 4 of those 64 isolates, the country of origin was undetermined. For the remaining three isolates, import status was unknown (*n*=1), or no connection to import (*n*=2) was revealed. Overall, we suspected 19 countries as potential origins (Fig. 1c), with Europe (30/60; 50%) and Asia (42/60; 39%) being the most represented regions. Thailand (*n*=11), Ukraine (*n*=11), Spain (*n*=8), India (*n*=5) and Greece (*n*=4) accounted for 65% of the isolates with known import countries. All isolates (*n*=11) from Ukrainian patients were identified in 2022, making up the majority of the 17 cases reported that year.

Isolates associated with Ukraine belonged to the genomic lineages ST1047 (*n*=5), ST773 (*n*=5) and ST664 (*n*=1) (Fig. 1c). Imports from several other countries as well encompassed different STs, including Thailand (STs 111, 235, 260, 357, 823, 941 and 1047), Spain (STs 111, 253, 313 and 348), India (STs 111, 316, 654, 773 and 1047), Greece (STs 111, 235 and 395) and Pakistan (STs 235, 308 and 654). On the other hand, all imports of ST253 (*n*=3; Spain), ST348 (*n*=3; Spain) and ST823 (*n*=3; Thailand) isolates were linked to one country.

The most prevalent STs in the collection were STs 235 (*n*=12), 111 (*n*=9) and 773 (*n*=9), comprising isolates originating from ≥5 more countries. Along with ST357 (*n*=3), ST233 (*n*=2), ST308 (*n*=1) and ST654 (*n*=2), they are among the top ten global high-risk clones [13]. Other globally spread STs associated with MDR and XDR phenotypes included ST253 (*n*=3), ST260 (*n*=1), ST395 (*n*=3), ST773 (*n*=9) and ST823 (*n*=3) [12,13]. These clonal lineages accounted for 72% of the Norwegian CP-Pa collection and included the two isolates with no import association (ST235 and ST348).

### Prevalence of specific carbapenemases and virulence factors

**Fig. 2.**
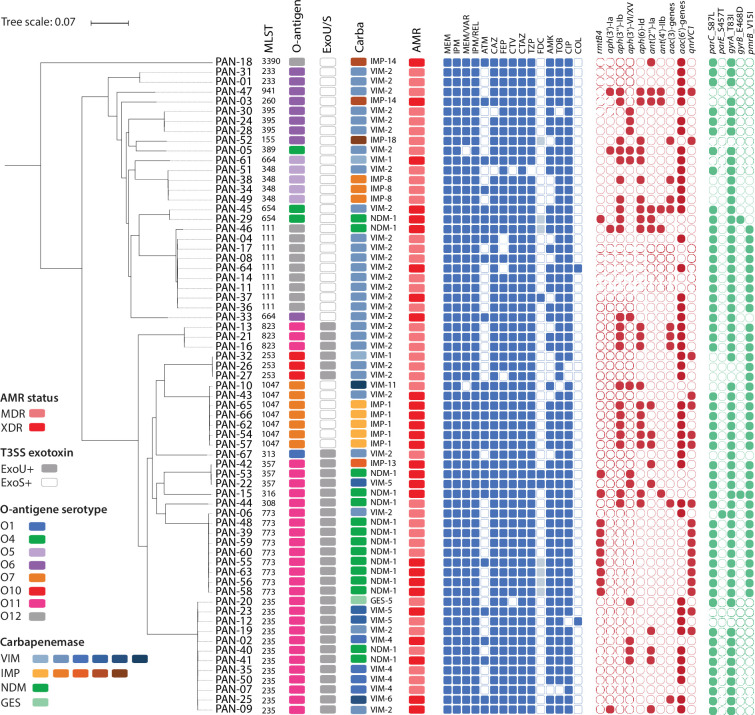
Phylogeny of the Norwegian collection of CP-Pa (PAN-01–PAN-67) with associated phenotypic and genotypic metadata. The rooted parsnp tree was constructed with a randomly selected reference genome (PAN-53), with a tree scale as indicated. The coupled metadata include MLST, O-antigen serotype, type 3 secretion system (T3SS) exotoxin (ExoU+ or ExoS+), carbapenemase (VIM, IMP, NDM and GES) variants and antimicrobial resistance (AMR) status (MDR or XDR) according to the shown colour codes. Additionally, the results from susceptibility testing against the indicated antipseudomonal antibiotics (squares coloured in blue, resistant; white, sensitive; or grey, area of technical uncertainty), the presence of acquired genes associated with resistance (red circle) to aminoglycosides (*rmtB4* and *aph*(3′)-, *aph*(3″)-, *aph*(6)-, *ant*(2″) and *ant*(4′)-variants) and ciprofloxacin (*qnrVC1*) and the presence of mutations (green circle) in quinolone-resistance determining regions (*gyrA/B* and *parC/E*) and mutations associated with resistance to colistin (*pmrB*_V15I) reported by AMRFinderPlus (https://www.ncbi.nlm.nih.gov/pathogens/antimicrobial-resistance/AMRFinder/) are illustrated. MEM, meropenem; IMP, imipenem; MEM/VAR, meropenem-vaborbactam; IMP/REL, imipenem-relebactam; ATM, aztreonam; CAZ, ceftazidime; FEP, cefepime; CTV, ceftazidime-avibactam; CTAZ, ceftolozane-tazobactam; TZP, piperacillin-tazobactam; FDC, cefiderocol; AMK, amikacin; TOB, tobramycin; CIP, ciprofloxacin; COL, colistin.

All isolates carried a single carbapenemase gene (Table S1), with the following distribution: *bla*_VIM_ (*n*=39; 58%), *bla*_NDM-1_ (*n*=15; 22%), *bla*_IMP_ (*n*=12; 18%) and *bla*_GES-5_ (*n*=1; 1.5%). The yearly distribution of carbapenemase variants is depicted in Fig. 1d. VIM variants comprised VIM-1 (*n*=2), VIM-2 (*n*=28), VIM-4 (*n*=4), VIM-5 (*n*=3), VIM-6 (*n*=1) and VIM-11 (*n*=1). The IMP-family carbapenemases included IMP-1 (*n*=5), IMP-8 (*n*=3), IMP-13 (*n*=1), IMP-14 (*n*=2) and IMP-18 (*n*=1). Of the main genetic lineages, ST111, ST773 and ST1047 were primarily associated with VIM-2, NDM-1 and IMP-1, respectively. In contrast, the carbapenemase content in ST235 was more variable with the presence of different VIM variants, NDM-1 or GES-5 (Fig. 2). We observed an increasing trend for NDM-1 since its first detection in 2013.

Several high-risk clones of *P. aeruginosa* are associated with the O-antigen serotype O11 [13], which was also found to be the most common O-antigen among the eight different types detected in our collection (Fig. 1e). The O11-positive isolates included STs 235, 301, 308, 316, 357, 773 and 823, accounting for 43% of the collection. Other prevalent serotypes were O12 (associated with STs 111 and 3390), O6 (associated with STs 155, 233, 260, 395, 663 and 941) and O7 (associated with ST1047). The type 3 secretion system (T3SS) exotoxin genes *exoU* and *exoS* were equally detected and explicitly linked to specific genetic lineages in the phylogenetic analyses (Fig. 2).

### Antimicrobial susceptibility testing

Susceptibility testing towards relevant antipseudomonal antibiotics (Table S1) categorized the isolates as MDR (*n*=39) or XDR (*n*=28) according to standard definitions [33]. The proportion of resistant isolates for each antibiotic, grouped by the carbapenemase family, is listed in Table 1. All isolates were resistant to carbapenems (meropenem and imipenem) as well as the *β*-lactam/*β*-lactamase inhibitor combinations imipenem-relebactam and ceftolozane-tazobactam. All except two isolates with VIM-2 or VIM-11 were resistant to meropenem-vaborbactam. Interestingly, they displayed a twofold lower MIC value for meropenem-vaborbactam. This rare phenotype has been associated with the AmpC R79Q mutation, which was not detected in our isolates, and the overexpression of MexXY and AmpC [34].

**Table 1.** Proportion of isolates resistant to the listed antipseudomonal antibiotics and proportions categorized as MDR or XDR according to carbapenemase family and among the overall Norwegian CP-Pa collection (2006–2022)

	VIM (*n*=39)	IMP (*n*=12)	NDM-1 (*n*=15)	GES-1 (*n*=1)	Overall (*n*=67)
Antibiotic	R* (%)	R (%)	R (%)	R (%)	R (%)
Meropenem	100	100	100	100	100
Meropenem/vaborbactam	95	100	100	100	97
Imipenem	100	100	100	100	100
Imipenem/relebactam	100	100	100	100	100
Aztreonam	31	75	40	0	40
Ceftazidime	95	100	100	100	97
Ceftazidime/avibactam	97	100	100	0	97
Cefepime	90	100	100	100	94
Ceftolozane/tazobactam	100	100	100	100	100
Piperacillin/tazobactam	97	100	100	100	97
Cefiderocol^†^	5	8	47	0	15
Amikacin	75	67	100	100	79
Tobramycin	92	100	100	100	96
Ciprofloxacin	92	100	100	100	96
Colistin	5	0	0	0	3
**Antimicrobial resistance category**	**No. of isolates (%)**	**No. of isolates (%)**	**No. of isolates (%)**	**No. of isolates (%)**	**No. of isolates (%)**
MDR	28 (72)	4 (33)	6 (40)	1 (100)	39 (58)
XDR	11 (28)	8 (67)	9 (60)	0	28 (42)

*Calculated from the number of isolates determined as resistant according to EUCAST Clinical Breakpoint Table v.14.0, 2024 (http://www.eucast.org.).

†Susceptibility testing was performed by the disc diffusion method with R including both the resistant and area of technical uncertainty (ATU) categories.

High overall resistance proportions were also found towards cephalosporins (ceftazidime and cefepime and ceftazidime-avibactam), piperacillin-tazobactam, fluoroquinolone (ciprofloxacin) and aminoglycosides (amikacin and tobramycin), with slightly lower rates towards amikacin in the VIM (75%) and IMP (67%) groups. Colistin (3%), cefiderocol (15%) and aztreonam (40%) exhibited the lowest overall resistance rates. The NDM-positive isolates had the highest proportion of resistance towards cefiderocol, with 7 out of 15 NDM isolates being resistant. However, six of the seven resistant isolates were found in the area of technical uncertainty category. IMP-positive isolates showed slightly higher proportions of aztreonam resistance (75%) compared to NDM-1 (40%) and VIM (31%) positives. The VIM group had the lowest proportions of resistance towards other tested antibiotics, except for colistin, where the resistant isolates (*n*=2) were VIM positive.

### Performance of phenotypic detection methods for carbapenemases

Carbapenemase activity was initially assessed using the *β*-CARBA test [35], which yielded positive results for all except seven VIM-2 and one VIM-4 encoding isolates (Table S1). To evaluate the clinical utility of other relevant methods for rapid carbapenemase detection, we compared *β*-CARBA results with those from IMI/IMD (combination gradient strip with imipenem and EDTA) and Coris O.K.N.V.I RESIST-5 test. The performance of these methods by the carbapenemase family is shown in Table 2. All three methods detected 100% of the NDM-1 isolates. *β*-CARBA and Coris tests detected all IMP isolates (*n*=12), whereas IMI/IMD was negative for 2 out of 12 isolates. For the detection of VIM-positive isolates (*n*=39), 92% of the isolates were positive with the Coris and IMI/IMD methods, while only 79% were positive with *β*-CARBA. The Coris test gave false negatives for the VIM-5 (*n*=3) variant, while *β*-CARBA failed to detect VIM-2 (7/28; 25%) and VIM-4 (1/4; 25%) positive isolates. As expected, the single GES carbapenemase isolate was only positive when tested by *β*-CARBA since the IMI/IMD and Coris tests are not designed to identify isolates with GES carbapenemases. Despite the high detection rates for all three methods [59/67 (88%) for *β*-CARBA, 61/67 (91%) for IMI/IMD and 63/66 (94%) for Coris], no single test was 100% reliable in detecting all CP-Pa in our collection.

**Table 2.** Phenotypical detection of carbapenemase and carbapenemase activity according to carbapenemase family

Carbapenemase family	No. of isolates (%) positive for the respective tests		
	*β*-CARBA*	IMI/IMD†	Coris O.K.N.V.I RESIST-5‡
VIM (*n*=39)	31 (79)	36 (92)	36 (92)
NDM-1 (*n*=15)	15 (100)	15 (100)	15 (100)
IMP (*n*=12)	12 (100)	10 (83)	12 (100)
GES-5 (*n*=1)	1 (100)	na	na

*Colorimetric test which detects carbapenemase production (Bernabeu *et al.*, 2017[35], doi: 10.1093/jac/dkx061).

†Combination strip with imipenem and EDTA which detects class B carbapenemases (metallo-*β*-lactamases).

‡Immunochromatography test which detects OXA-48-like, KPC, NDM, VIM and IMP.

na, not applicable.

### Genetic characteristics and population structure of the Norwegian CP-Pa collection

The parsnp phylogeny and all genotypic and phenotypic data (Fig. 2) confirmed the extensive genomic diversity of the CP-Pa collection, consistent with identifying 21 STs. The phylogenetic tree showed 11 distinct clusters (≥2 genomes each) where genomes share the same ST, O-antigen serotype and T3SS exotoxin (ExoU or ExoS). However, several clusters contained two or more carbapenemase variants, as follows: VIM-2 and NDM-1 (ST111); VIM-2, -4, -5 and -6 and GES-5 and NDM-1 (ST235); VIM-2 and IMP-8 (ST348); VIM-1 and VIM-2 (ST253); VIM-5, IMP-13 and NDM-1 (ST357); VIM-2 and NDM-1 (ST654); VIM-2 and NDM-1 (ST773); and VIM-2, VIM-11 and IMP-1 (ST1047). The carbapenemase heterogeneity within specific clusters implies several horizontal acquisition events, consistent with previous reports on endemic clones associated with MDR/XDR phenotypes [12]. Although antimicrobial resistance categories and resistance patterns, including additional acquired resistance genes and chromosomal mutations, generally followed the ST profile, some heterogeneity was observed, particularly within the ST235, ST348, ST357 and ST1047 clusters (Fig. 2, Table S1).

The high proportion of aminoglycoside resistance observed among the isolates correlated with the presence of genes encoding 16S RNA methylase (*rmtB4*) and various AMEs. The *rmtB4* gene was primarily found in the ST773 (NDM-1) clone, while the other clusters encoded different combinations of AMEs. Variants of all three main classes of AME, phosphotransferase (*aph*), nucleotidyltransferase (*ant*) and acetyltransferase (*acc*) genes, were identified in most isolates. The ST1047 (IMP-1) isolates encoded four AME genes, representing all three classes.

Resistance to ciprofloxacin correlated with mutations in the quinolone-resistance-determining regions of *gyrA* and *parC* [36] detected in most isolates (62/67). Two of the five WT *gyrA* and *parC* isolates carried *qnrVC1* and were resistant to ciprofloxacin (Table S1). Except for one isolate with *qnrA*, *qnrVC1* (*n*=18) was the only acquired fluoroquinolone resistance determinant detected in our collection. Q*nrVC1* was detected in all ST773 (NDM-1) cluster isolates and had an increasing trend since its first detection in 2014.

Two isolates, PAN-12 (ST235) and PAN-64 (ST111) exhibited resistance to colistin (MIC: 8 mg l^−1^). No specific genetic markers associated with colistin resistance were identified. In the latter isolate, AMRFinderPlus reported a nonsynonymous mutation (V15I) in the *pmrB* gene, part of a two-component system regulating LPS modification and mediating colistin resistance [37]. However, this mutation was present in several other isolates (*n*=35) and could not be directly linked to increased resistance to colistin. A comparison of the PmrB aa sequence from PAN-12 and PAN-64 with PAO1 (NC_002516.2) and PA14 (NC_008463.1) revealed a higher identity to the PA14 protein, which differed from PAO1 in five positions, including V to I in position 15. In PAN-64, the only difference from PA14 was the nonsynonymous mutation V6A. PAN-12 exhibited two nonsynonymous mutations compared to PA14, I15V (identical to PAO1) and G362S. The two isolates had no mutations related to colistin resistance in *phoPQ*, encoding another two-component system necessary for LPS modification and resistance to polymyxins [38].

Using the disc diffusion method, susceptibility testing towards cefiderocol revealed three resistant isolates in our collection: PAN-22, PAN-37 and PAN-53. Resistance to cefiderocol is associated with a combination of mechanisms, including chromosomal mutations [39]. However, we did not detect any previously described mutations leading to cefiderocol resistance in the TonB-dependent siderophore receptors PiuD, FtpA and PirA, including the regulator PirR [40,42] and AmpC [42].

### Genetic relatedness and evaluation of regional dissemination of CP-Pa

**Fig. 3.**
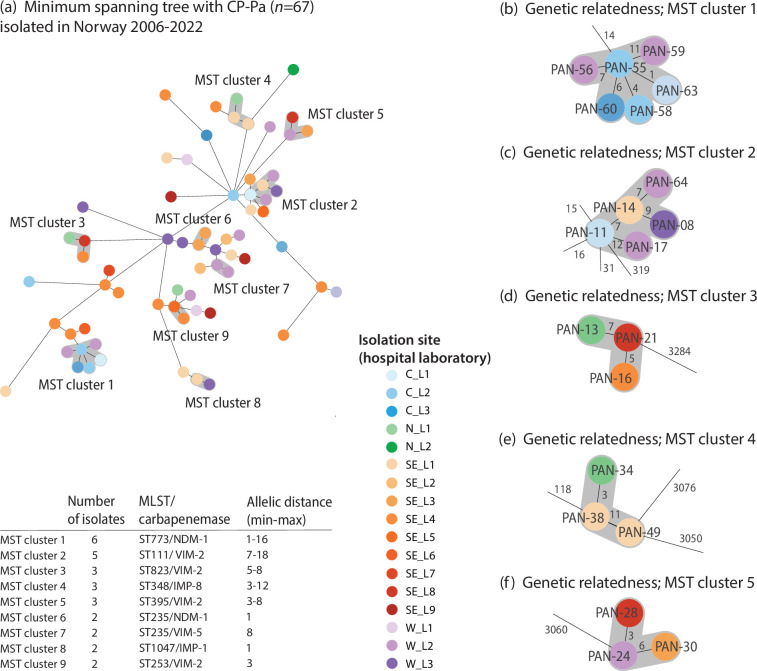
Genetic relatedness between the CP-Pa (PAN-01–PAN-67) isolated at various locations in Norway. (a) MST with distance based on 3867 cg alleles and cluster (grey shading) threshold ≤12 allelic differences and (b–e) focusing on specific clusters (≥3 genomes, node distances indicated) within the tree. Each node represents one genome with colouring according to the isolation site, with codes reflecting individual hospital laboratories in the four health regions (C, central; N, north; SE, south east; and W, west).

To investigate the potential dissemination of CP-Pa among Norwegian patients, we examined genetic relatedness within our collection using cgMLST phylogeny. The MST (Fig. 3a) revealed nine clusters (MST clusters 1–9), suggesting potential transmission events. The clusters featured distinct ST/carbapenemase combinations and included varying numbers of isolates (Fig. 3a–f).

As reflected by the node colours, MST clusters 1, 2, 4, 6 and 7 included two isolates from the same hospital laboratories. However, additional metadata (Table S1) showed that PAN-17 and PAN-64 (MST cluster 2), PAN-38 and PAN-49 (MST cluster 4) and PAN-12 and PAN-23 (MST cluster 7) were isolated 3 to 9 years apart, ruling out a direct epidemiological link. On the other hand, PAN-40 and PAN-41 (MST cluster 6) were sampled 1 month apart. The first was linked to import (country unknown), while the latter had no association with travel abroad, suggesting a possible domestic transmission event within the same hospital.

In MST clusters 1 and 8, the isolates were epidemiologically linked by association with patients previously hospitalized in Ukraine in 2022. However, domestic patient-to-patient transmissions cannot be ruled out. Within MST cluster 1, the same laboratory isolated PAN-55 and PAN-56 (C_L2) and PAN-58 and PAN-59 (W_L2), which had 16 and 4 allelic differences, respectively.

Other MST clusters with isolates linked to a single import country included MST cluster 3 (Thailand), MST cluster 4 (Spain), cluster 5 (Greece), MST cluster 7 (Turkey) and MST cluster 9 (Spain). Each of these clusters contained isolates from different hospital laboratories. The time between the two MST cluster 5 isolates was 3 months, and the time between the cluster 9 isolates was 1 month, suggesting transmission in Greece and Spain, respectively, before admission to healthcare in Norway. Within the other clusters, the timespan varied from 1 to 4 years, with a notable 12 years for ST111.

## Discussion

CP-Pa is increasing globally, yet Norway remains a low-prevalence country. Between 2006 and 2022, the annual number of reported CP-Pa cases in Norway ranged from 1 to 17, with an increasing trend confirmed by the 2023 numbers (*n*=26) [43]. Most cases were sporadic and linked to international travel or hospital transfers from abroad. Of concern, Norwegian CP-Pa isolates exhibited MDR or XDR phenotypes, highlighting a significant clinical problem.

The CP-Pa collection displayed high proportions of resistance towards all relevant antipseudomonal antibiotics tested, including the *β*-lactam/*β*-lactamase inhibitor combinations ceftolozane-tazobactam, piperacillin-tazobactam, ceftazidime-avibactam, imipenem-relebactam and meropenem-vaborbactam. This significantly limits the available therapeutic options. Based on our susceptibility testing results, only colistin, the siderophore-cephalosporin cefiderocol [44], and partly aztreonam currently appear as relevant antibiotics for treating CP-Pa infections in Norway. Promising novel *β*-lactam/*β*-lactamase inhibitor combinations that might result in additional therapeutic choices are undergoing clinical trials [45,46]. The newly approved aztreonam/avibactam combination might be an alternative, but recent extensive activity surveys have shown susceptibility ranges around 80%, comparable to piperacillin/tazobactam [47]. However, clinical data for treatment against CP-Pa and availability will be crucial for future medical use.

Our analyses of genetic relationships revealed multiple clusters of well-known global high-risk clones and other emerging international lineages [12,13]. Several of these clusters contained highly related genomes, indicating possible transmission events. However, only two isolates were closely connected epidemiologically regarding time and location, pointing to potential patient-to-patient transfer in a Norwegian hospital. This underscores the importance of relevant metadata for distinguishing between repeated imports and domestic spread or outbreaks. Effective national infection control systems and screening patients with risk factors may explain the low incidence of domestic transmission of CP-Pa in Norway.

Since the summer of 2022, Norway has experienced an increase in XDR CP-Pa linked to the medical evacuation of seriously injured war victims from Ukraine. Other European countries have also reported a rise in carbapenemase-producing Gram-negative bacteria associated with imports from Ukraine [21,48,50]. Furthermore, the NDM-1 (ST773) and IMP-1 (ST1047) *P. aeruginosa* clones identified in Norway have been isolated from Ukrainian patients in other European countries, including Denmark, Germany, Spain and the Netherlands [21,48, 51, 52].

The ST773 lineage was of O-antigen serotype 11 and *exoU*+, consistent with characteristics of prominent global high-risk clones, including ST235, ST357, ST308 and ST298 [12]. The expression of the ExoU virulence factor in NDM-positive *P. aeruginosa* ST308 has previously been associated with increased mortality in cases of bacteraemia [53]. Additionally, the NDM-ST773 isolates encoded the RmtB4 16S RNA methylase and the fluoroquinolone resistance determinant QnrVC1, rendering them non-susceptible to all tested antimicrobials except for cefiderocol and colistin. These isolates also expressed either resistance or susceptible increased exposure (WT) to aztreonam, with a similar antibiogram reported for an NDM-ST733 isolate from Spain [52]. The Norwegian ST773 isolates were linked to imports from five countries, with one encoding VIM-2 and the rest NDM-1. These characteristics strongly support the notion that ST773 belongs to the globally expanding high-risk lineages [13,54, 55], with the further worrisome capability of disseminating NDM-1 through *P. aeruginosa,* as previously addressed [52].

In conclusion, this study demonstrates that infections and colonization with CP-Pa in Norway are caused by high-risk global MDR/XDR clones associated with international travel or hospitalization abroad, with limited treatment options. To address the emergence of these worrisome clones, a comprehensive approach integrating surveillance, infection control, antimicrobial stewardship and research efforts is crucial to prevent and contain further dissemination. In countries with low incidence rates like Norway, improved genomic surveillance and advanced molecular typing methods are essential for distinguishing between imported cases and local transmission events. This enables timely and appropriate infection control measures. However, the global nature of the problem emphasizes the need for international collaboration in monitoring and controlling the spread of CP-Pa.

## Supplementary material

10.1099/jmm.0.001944 Uncited Table S1.

## Abbreviations

AME: aminoglycoside-modifying enzyme
cg: core genome
CP-Pa: carbapenemase-producing *Pseudomonas aeruginosa*
GES: Guiana extended spectrum metallo-*β*-lactamase
IMP: imipenemase metallo-*β*-lactamase
MDR: multidrug-resistant
MLST: multilocus sequence type
MSIS: The Norwegian Surveillance System for Communicable Diseases
MST: minimum spanning tree
NDM: New Delhi metallo-*β*-lactamase
ST: sequence type
T3SS: type 3 secretion system
VIM: Verona integron-encoded metallo-*β*-lactamase
WGS: whole-genome sequencing
XDR: extensively drug-resistant
